# 1,3-Dihy­droxy-2-(hy­droxy­meth­yl)propan-2-aminium formate

**DOI:** 10.1107/S1600536811015534

**Published:** 2011-04-29

**Authors:** Guo-Bin Ren, Ming-Hui Qi, Jin-Yao Chen, Kun-Yan Meng, Jia-Liang Zhong

**Affiliations:** a Pharmaceutical Crystal Engineering Research Group, Shanghai Institute of Pharmaceutical Industry, 1320 Beijing Road (W), Shanghai 200040, People’s Republic of China

## Abstract

The title compound, C_4_H_12_NO_3_^+^·CHO_2_^−^, was obtained from 1,3-dihy­droxy-2-(hy­droxy­meth­yl)propan-2-aminium acetate and ethyl formate. In the crystal, the cations and anions are held together by inter­molecular N—H⋯O and O—H⋯O hydrogen bonds.

## Related literature

For background to the use of tris­(hy­droxy­meth­yl)amino­methane in biochemistry and mol­ecular biology, see: Gomori (1955 ▶). For related structrues, see: Stepniak *et al.* (2003 ▶); Yu & Qian (2009 ▶).
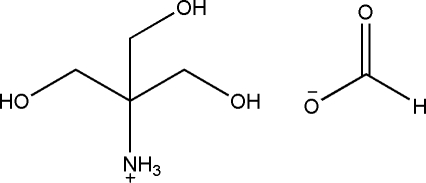


## Experimental

### 

#### Crystal data

C_4_H_12_NO_3_^+^·CHO_2_^−^
*M* *_r_* = 167.16
Orthorhombic, 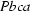

*a* = 6.4980 (1) Å
*b* = 11.8740 (1) Å
*c* = 20.5897 (2) Å
*V* = 1588.64 (3) Å^3^
*Z* = 8
Cu *K*α radiation
μ = 1.08 mm^−1^
*T* = 296 K
0.23 × 0.18 × 0.10 mm

#### Data collection

Bruker APEXII diffractometer
Absorption correction: multi-scan (*SADABS*; Bruker, 2005 ▶) *T*_min_ = 0.789, *T*_max_ = 0.899
4402 measured reflections
1368 independent reflections
1304 reflections with *I* > 2σ(*I*)
*R*_int_ = 0.016

#### Refinement

*R*[*F* ^2^ > 2σ(*F* ^2^)] = 0.034
*wR*(*F* ^2^) = 0.095
*S* = 1.09
1368 reflections
117 parameters
H atoms treated by a mixture of independent and constrained refinement
Δρ_max_ = 0.28 e Å^−3^
Δρ_min_ = −0.20 e Å^−3^

### 

Data collection: *APEX2* (Bruker, 2005 ▶); cell refinement: *SAINT* (Bruker, 2005 ▶); data reduction: *SAINT*; program(s) used to solve structure: *SHELXS97* (Sheldrick, 2008 ▶); program(s) used to refine structure: *SHELXL97* (Sheldrick, 2008 ▶); molecular graphics: *SHELXTL* (Sheldrick, 2008 ▶); software used to prepare material for publication: *SHELXL97*.

## Supplementary Material

Crystal structure: contains datablocks I, global. DOI: 10.1107/S1600536811015534/cv5073sup1.cif

Structure factors: contains datablocks I. DOI: 10.1107/S1600536811015534/cv5073Isup2.hkl

Supplementary material file. DOI: 10.1107/S1600536811015534/cv5073Isup3.cml

Additional supplementary materials:  crystallographic information; 3D view; checkCIF report

## Figures and Tables

**Table 1:** Hydrogen-bond geometry (Å, °)

*D*—H⋯*A*	*D*—H	H⋯*A*	*D*⋯*A*	*D*—H⋯*A*
O1—H1*A*⋯O4	0.82	1.92	2.7378 (14)	175
O2—H2*A*⋯O3^i^	0.82	1.87	2.6845 (12)	173
O3—H3*A*⋯O5^ii^	0.82	1.85	2.6659 (14)	180
N1—H1*B*⋯O2^iii^	0.918 (18)	1.933 (19)	2.8233 (14)	163.0 (15)
N1—H1*C*⋯O4^ii^	0.960 (18)	1.861 (18)	2.8171 (15)	173.4 (15)
N1—H1*D*⋯O5^iv^	0.946 (18)	1.857 (19)	2.7876 (14)	167.2 (15)

Symmetry codes: (i) 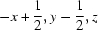; (ii) 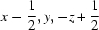; (iii) 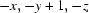; (iv) 
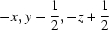
.

**Fig. 1.**
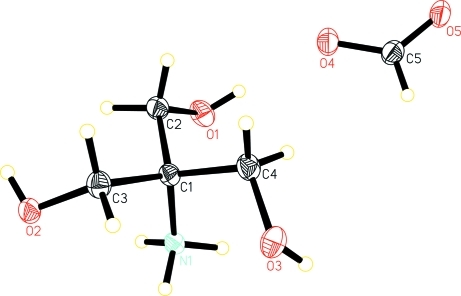
The content of asymmetric unit of (I) showing the atom-labelling scheme. Displacement ellipsoids are drawn at the 30% probability level.

**Fig. 2.**
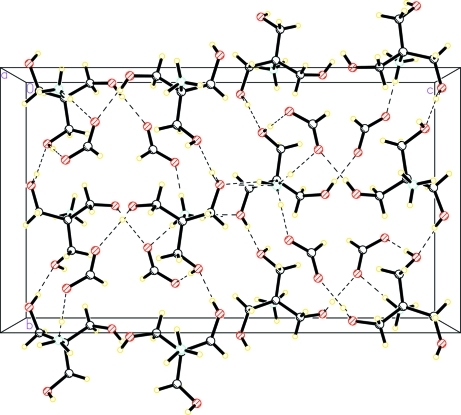
The packing of the title compound, viewed down the a axis. The dashed lines indicate the hydrogen bonds.

## supplementary crystallographic information

### Comment

Tris(hydroxymethyl)aminomethane (*Tris*) is extensively used in biochemistry and molecular biology (Gomori, 1955). In biochemistry, *Tris* is widely used as a component of buffer solutions. In this paper, we report the crystal structure of its formate salt - the title compound (I).

The structure of (I) is built up from cations and anions (Fig. 1) connected through strong intermolecular hydrogen bonds (Table 1, Fig. 2). The bond lengths and angles in the molecule are normal and comparable with those observed in the related compounds (Stepniak *et al.*, 2003; Yu *et al.*, 2009).

### Experimental

Suitable X-ray crystals of the title compound was obtained by dissolving 1,3-dihydroxy-2-(hydroxymethyl)propan-2-aminium asiatate in ethyl formate, and standing overnight at room temperature.

### Refinement

The formic acid and N-bound H atoms located in a difference Fourier map and isotropically refined. All others H atoms were geometrically positioned [C—H 0.97 Å; O—H 0.82 Å] and refined as riding with *U*_iso_(H) = 1.2-1.5 *U*_eq_of the parent atom.

### Crystal data

**Table:**

C_4_H_12_NO_3_^+^·CHO_2_^−^	*F*(000) = 720
*M**_r_* = 167.16	*D*_x_ = 1.398 Mg m^−^^3^
Orthorhombic, *P**b**c**a*	Cu *K*α radiation, λ = 1.54178 Å
Hall symbol: -P 2ac 2ab	Cell parameters from 3109 reflections
*a* = 6.4980 (1) Å	θ = 7.5–66.8°
*b* = 11.8740 (1) Å	µ = 1.08 mm^−^^1^
*c* = 20.5897 (2) Å	*T* = 296 K
*V* = 1588.64 (3) Å^3^	Block, colourless
*Z* = 8	0.23 × 0.18 × 0.10 mm

### Data collection

**Table:**

Bruker APEXII diffractometer	1368 independent reflections
Radiation source: fine-focus sealed tube	1304 reflections with *I* > 2σ(*I*)
graphite	*R*_int_ = 0.016
Detector resolution: 0 pixels mm^-1^	θ_max_ = 67.3°, θ_min_ = 7.5°
φ and ω scans	*h* = −7→7
Absorption correction: multi-scan (*SADABS*; Bruker, 2005)	*k* = −13→13
*T*_min_ = 0.789, *T*_max_ = 0.899	*l* = −22→24
4402 measured reflections	

### Refinement

**Table:**

Refinement on *F*^2^	Secondary atom site location: difference Fourier map
Least-squares matrix: full	Hydrogen site location: inferred from neighbouring sites
*R*[*F*^2^ > 2σ(*F*^2^)] = 0.034	H atoms treated by a mixture of independent and constrained refinement
*wR*(*F*^2^) = 0.095	*w* = 1/[σ^2^(*F*_o_^2^) + (0.055*P*)^2^ + 0.4117*P*] where *P* = (*F*_o_^2^ + 2*F*_c_^2^)/3
*S* = 1.09	(Δ/σ)_max_ < 0.001
1368 reflections	Δρ_max_ = 0.28 e Å^−^^3^
117 parameters	Δρ_min_ = −0.20 e Å^−^^3^
0 restraints	Extinction correction: *SHELXL97* (Sheldrick, 2008), Fc^*^=kFc[1+0.001xFc^2^λ^3^/sin(2θ)]^-1/4^
Primary atom site location: structure-invariant direct methods	Extinction coefficient: 0.0049 (6)

### Special details

**Table:**

Geometry. All e.s.d.'s (except the e.s.d. in the dihedral angle between two l.s. planes) are estimated using the full covariance matrix. The cell e.s.d.'s are taken into account individually in the estimation of e.s.d.'s in distances, angles and torsion angles; correlations between e.s.d.'s in cell parameters are only used when they are defined by crystal symmetry. An approximate (isotropic) treatment of cell e.s.d.'s is used for estimating e.s.d.'s involving l.s. planes.
Refinement. Refinement of *F*^2^ against ALL reflections. The weighted *R*-factor *wR* and goodness of fit *S* are based on *F*^2^, conventional *R*-factors *R* are based on *F*, with *F* set to zero for negative *F*^2^. The threshold expression of *F*^2^ > σ(*F*^2^) is used only for calculating *R*-factors(gt) *etc*. and is not relevant to the choice of reflections for refinement. *R*-factors based on *F*^2^ are statistically about twice as large as those based on *F*, and *R*- factors based on ALL data will be even larger.

### Fractional atomic coordinates and isotropic or equivalent isotropic displacement parameters (Å^2^)

**Table:**

	*x*	*y*	*z*	*U*_iso_*/*U*_eq_	
O1	0.12876 (16)	0.52355 (9)	0.22191 (4)	0.0392 (3)	
H1A	0.1666	0.5765	0.2445	0.059*	
O2	0.20496 (15)	0.43932 (8)	0.02376 (4)	0.0340 (3)	
H2A	0.2808	0.3926	0.0407	0.051*	
O3	0.06605 (15)	0.77248 (7)	0.07395 (5)	0.0349 (3)	
H3A	−0.0395	0.7945	0.0917	0.052*	
N1	−0.06449 (16)	0.55746 (9)	0.10599 (5)	0.0254 (3)	
C1	0.16134 (18)	0.58258 (10)	0.10896 (6)	0.0251 (3)	
C2	0.2530 (2)	0.51726 (11)	0.16588 (6)	0.0322 (3)	
H2B	0.2694	0.4389	0.1535	0.039*	
H2C	0.3883	0.5472	0.1758	0.039*	
C3	0.2620 (2)	0.54863 (11)	0.04459 (6)	0.0308 (3)	
H3B	0.2234	0.6026	0.0114	0.037*	
H3C	0.4103	0.5516	0.0495	0.037*	
C4	0.1855 (2)	0.70956 (11)	0.11900 (7)	0.0318 (3)	
H4A	0.1435	0.7288	0.1628	0.038*	
H4B	0.3293	0.7298	0.1142	0.038*	
O4	0.23553 (18)	0.69672 (9)	0.30298 (5)	0.0481 (3)	
O5	0.22296 (18)	0.84393 (9)	0.36823 (5)	0.0454 (3)	
C5	0.1807 (2)	0.79327 (12)	0.31701 (7)	0.0360 (4)	
H5A	0.098 (3)	0.8304 (16)	0.2871 (8)	0.051 (5)*	
H1B	−0.120 (3)	0.5727 (13)	0.0659 (9)	0.040 (4)*	
H1C	−0.142 (2)	0.6018 (15)	0.1363 (8)	0.041 (4)*	
H1D	−0.098 (3)	0.4814 (16)	0.1148 (8)	0.044 (4)*	

### Atomic displacement parameters (Å^2^)

**Table:**

	*U*^11^	*U*^22^	*U*^33^	*U*^12^	*U*^13^	*U*^23^
O1	0.0514 (6)	0.0413 (6)	0.0248 (5)	−0.0045 (5)	−0.0007 (4)	−0.0006 (4)
O2	0.0468 (6)	0.0275 (5)	0.0277 (5)	0.0099 (4)	−0.0082 (4)	−0.0036 (3)
O3	0.0403 (6)	0.0239 (5)	0.0406 (6)	0.0002 (4)	0.0064 (4)	0.0060 (4)
N1	0.0302 (6)	0.0215 (5)	0.0244 (6)	−0.0025 (4)	−0.0019 (4)	0.0015 (4)
C1	0.0274 (6)	0.0227 (6)	0.0253 (6)	−0.0020 (5)	−0.0008 (5)	−0.0011 (5)
C2	0.0378 (7)	0.0317 (7)	0.0272 (7)	0.0042 (5)	−0.0046 (5)	−0.0019 (5)
C3	0.0352 (7)	0.0297 (7)	0.0276 (7)	0.0003 (5)	0.0027 (5)	−0.0001 (5)
C4	0.0354 (7)	0.0239 (7)	0.0360 (7)	−0.0044 (5)	−0.0009 (5)	−0.0019 (5)
O4	0.0633 (7)	0.0367 (6)	0.0444 (6)	0.0148 (5)	−0.0142 (5)	−0.0112 (4)
O5	0.0551 (7)	0.0359 (6)	0.0451 (6)	0.0153 (5)	−0.0088 (5)	−0.0116 (4)
C5	0.0388 (8)	0.0327 (7)	0.0366 (8)	0.0069 (6)	−0.0024 (6)	−0.0001 (6)

### Geometric parameters (Å, °)

**Table:**

O1—C2	1.4100 (16)	C1—C4	1.5299 (16)
O1—H1A	0.8200	C1—C3	1.5317 (17)
O2—C3	1.4163 (16)	C2—H2B	0.9700
O2—H2A	0.8200	C2—H2C	0.9700
O3—C4	1.4217 (16)	C3—H3B	0.9700
O3—H3A	0.8200	C3—H3C	0.9700
N1—C1	1.4987 (15)	C4—H4A	0.9700
N1—H1B	0.918 (18)	C4—H4B	0.9700
N1—H1C	0.960 (18)	O4—C5	1.2348 (18)
N1—H1D	0.946 (18)	O5—C5	1.2449 (18)
C1—C2	1.5265 (17)	C5—H5A	0.928 (19)
			
C2—O1—H1A	109.5	O1—C2—H2C	109.2
C3—O2—H2A	109.5	C1—C2—H2C	109.2
C4—O3—H3A	109.5	H2B—C2—H2C	107.9
C1—N1—H1B	112.4 (11)	O2—C3—C1	113.05 (10)
C1—N1—H1C	112.2 (10)	O2—C3—H3B	109.0
H1B—N1—H1C	105.7 (14)	C1—C3—H3B	109.0
C1—N1—H1D	113.9 (10)	O2—C3—H3C	109.0
H1B—N1—H1D	105.7 (14)	C1—C3—H3C	109.0
H1C—N1—H1D	106.2 (14)	H3B—C3—H3C	107.8
N1—C1—C2	108.19 (10)	O3—C4—C1	111.94 (10)
N1—C1—C4	107.59 (10)	O3—C4—H4A	109.2
C2—C1—C4	110.91 (10)	C1—C4—H4A	109.2
N1—C1—C3	109.28 (10)	O3—C4—H4B	109.2
C2—C1—C3	111.35 (10)	C1—C4—H4B	109.2
C4—C1—C3	109.43 (10)	H4A—C4—H4B	107.9
O1—C2—C1	112.20 (10)	O4—C5—O5	125.67 (14)
O1—C2—H2B	109.2	O4—C5—H5A	116.9 (12)
C1—C2—H2B	109.2	O5—C5—H5A	117.4 (11)
			
N1—C1—C2—O1	−43.81 (13)	C4—C1—C3—O2	−165.66 (10)
C4—C1—C2—O1	73.96 (13)	N1—C1—C4—O3	−49.91 (13)
C3—C1—C2—O1	−163.92 (11)	C2—C1—C4—O3	−168.05 (10)
N1—C1—C3—O2	−48.09 (14)	C3—C1—C4—O3	68.72 (13)
C2—C1—C3—O2	71.38 (14)		

### Hydrogen-bond geometry (Å, °)

**Table:**

*D*—H···*A*	*D*—H	H···*A*	*D*···*A*	*D*—H···*A*
O1—H1A···O4	0.82	1.92	2.7378 (14)	175
O2—H2A···O3^i^	0.82	1.87	2.6845 (12)	173
O3—H3A···O5^ii^	0.82	1.85	2.6659 (14)	180
N1—H1B···O2^iii^	0.918 (18)	1.933 (19)	2.8233 (14)	163.0 (15)
N1—H1C···O4^ii^	0.960 (18)	1.861 (18)	2.8171 (15)	173.4 (15)
N1—H1D···O5^iv^	0.946 (18)	1.857 (19)	2.7876 (14)	167.2 (15)

Symmetry codes: (i) −*x*+1/2, *y*−1/2, *z*; (ii) *x*−1/2, *y*, −*z*+1/2; (iii) −*x*, −*y*+1, −*z*; (iv) −*x*, *y*−1/2, −*z*+1/2.

## References

[bb1] Bruker (2005). *SADABS*, *APEX2* and *SAINT* Bruker AXS Inc., Madison, Wisconsin, USA.

[bb2] Gomori, G. (1955). *Methods in Enzymology*, Vol. 1, edited by S. P. Colowick & N. O. Kaplan, pp. 138–146. New York: Academic Press.

[bb3] Sheldrick, G. M. (2008). *Acta Cryst.* A**64**, 112–122. 10.1107/S0108767307043930 18156677

[bb4] Stepniak, K., Lis, T. & Koziol, A. E. (2003). *Z. Kristallogr. New Cryst. Struct.* **218**, 37–38.

[bb5] Yu, Y.-H. & Qian, K. (2009). *Acta Cryst.* E**65**, o1278. 10.1107/S1600536809016626 PMC2969592 21583140

